# Role of Serum HE4, Selected Clinical Features and Diagnostic Prediction Models in Differentiating Benign and Malignant Endometrial Lesions in Polish Women: A Prospective Cohort Study

**DOI:** 10.3390/cancers18213506

**Published:** 2026-10-30

**Authors:** Kamila Krejczy, Jakub Rzepka, Olga Wiśniewska, Kaja Michalczyk, Anita Chudecka-Głaz, Rafał Rzepka

**Affiliations:** 1 Department of Gynecology and Obstetrics, University of Zielona Gora, Zyty 26, 65046 Zielona Gora, Poland; 2 Department of Gynecological Surgery and Gynecological Oncology of Adults and Adolescents, Pomeranian Medical University, 70-204 Szczecin, Poland

**Keywords:** endometrial cancer, endometrial malignancy, HE4, REM score, Model A, endometrial thickness, abnormal uterine bleeding

## Abstract

Background/Objectives: The differentiation of endometrial cancer from non-malignant endometrial findings remains clinically important. We evaluated serum human epididymis protein 4 (HE4), clinical and ultrasound features, and two published diagnostic models, the Risk of Endometrial Malignancy (REM) score and Model A. Methods: In this prospective cohort, 110 women with abnormal uterine bleeding and/or ultrasound abnormalities underwent HE4 testing, transvaginal ultrasonography, and hysteroscopy with histopathological assessment. Original univariable analyses were supplemented by reanalysis of the individual study records, including ROC metrics, paired comparisons of AUCs, and an exploratory logistic model adjusting for age and endometrial thickness. Results: Endometrial cancer was diagnosed in 18 women; 92 had non-malignant findings. Median HE4 was higher in the cancer group (118.35 versus 52.3 pmol/L; *p* < 0.001, as originally reported). The AUC (95% bootstrap CI) was 0.834 (0.686–0.951) for HE4, 0.905 (0.794–0.981) for REM, and 0.840 (0.702–0.948) for Model A. AUC differences were not statistically significant (paired DeLong tests, all *p* > 0.10). In an exploratory adjusted analysis, each doubling of HE4 was associated with higher odds of malignancy (adjusted OR 5.41; 95% CI 1.81–16.21; *p* = 0.0025). Conclusions: HE4 and both published models discriminated between malignant and non-malignant findings in this selected cohort. The findings are exploratory and require independent validation before clinical use.

## Simple Summary

Endometrial cancer is one of the most common gynecological malignancies. We examined whether a blood test for human epididymis protein 4 (HE4), alone or combined with clinical and ultrasound findings, could help distinguish cancer from non-malignant endometrial conditions. In this prospective study of 110 women undergoing endometrial assessment, 18 had endometrial cancer. HE4 concentrations were higher in women with cancer. Three approaches—HE4 alone, the Risk of Endometrial Malignancy (REM) score, and Model A—were compared. REM had the highest numerical accuracy, although the differences between the three approaches were not statistically significant. In an exploratory analysis, HE4 remained associated with cancer after accounting for age and endometrial thickness. These findings require confirmation in larger, independent groups before the tests can be used to guide clinical decisions.

## 1. Introduction

Endometrial cancer is among the most common gynecological malignancies, particularly in countries with a high Human Development Index (HDI). Its incidence is influenced by demographic and lifestyle-related factors, including obesity, type 2 diabetes, and hypertension [1,2,3,4,5,6,7,8,9,10,11]. This growing burden reinforces the need for timely, reliable assessment of women who present with abnormal uterine bleeding or suspicious endometrial findings on ultrasound.

Early diagnosis is important, but no serum marker is currently recommended for population screening for endometrial cancer. Abnormal bleeding, especially after menopause, remains a key indication for endometrial investigation, and histopathology is required to confirm or exclude malignancy [12]. Therefore, the potential role of a blood-based marker is not to replace tissue diagnosis, but to support risk stratification in women already referred for endometrial evaluation.

Serum CA125 and human epididymis protein 4 (HE4) have been investigated as potential biomarkers in endometrial cancer [13,14,15]. HE4 is a secreted glycoprotein encoded by WFDC2 and belongs to a family of proteins containing a whey acidic protein four-disulfide-core domain. HE4 is also used in the assessment of ovarian malignancy and is expressed in several other cancer types, including lung and breast cancers [16]. Its serum concentration is therefore not specific for endometrial cancer; age and impaired renal function may also increase measured values. Experimental investigations have implicated HE4 in tumor-cell proliferation and invasion, although the exact pathways remain incompletely defined. Higher HE4 levels have been associated with more advanced endometrial cancer and deeper myometrial invasion [17,18]. The present investigation evaluates its diagnostic association rather than its biological mechanism.

**Figure 1:**
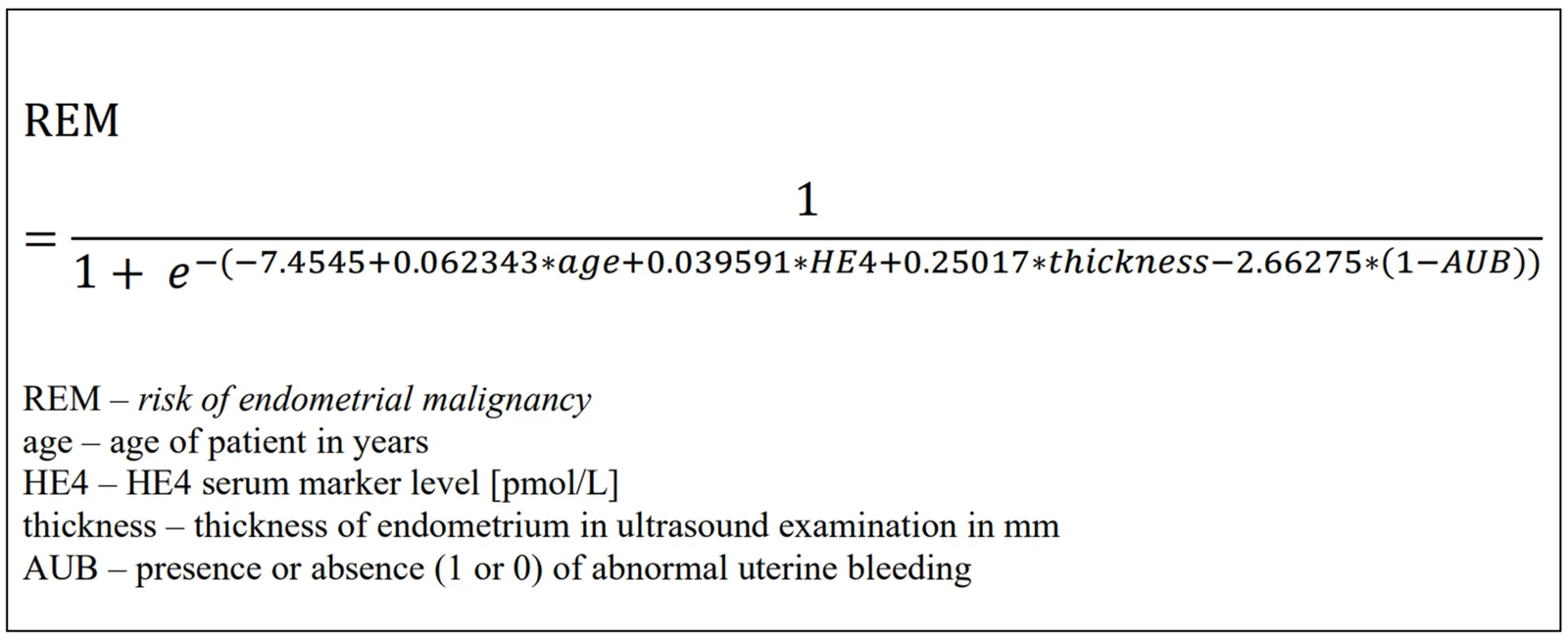
Risk of Endometrial Malignancy.

In 2013, Angioli et al. proposed the Risk of Endometrial Malignancy (REM) score [19]. This published equation combines abnormal uterine bleeding, age, serum HE4, and sonographic endometrial thickness (Figure 1). Its originally reported sensitivity and specificity were 92% and 96%, respectively, in the development population. REM was developed for endometrial malignancy, whereas the Risk of Ovarian Malignancy Algorithm (ROMA) is intended for ovarian malignancy risk assessment and is not a directly comparable endometrial cancer diagnostic tool [20,21].

**Figure 2:**
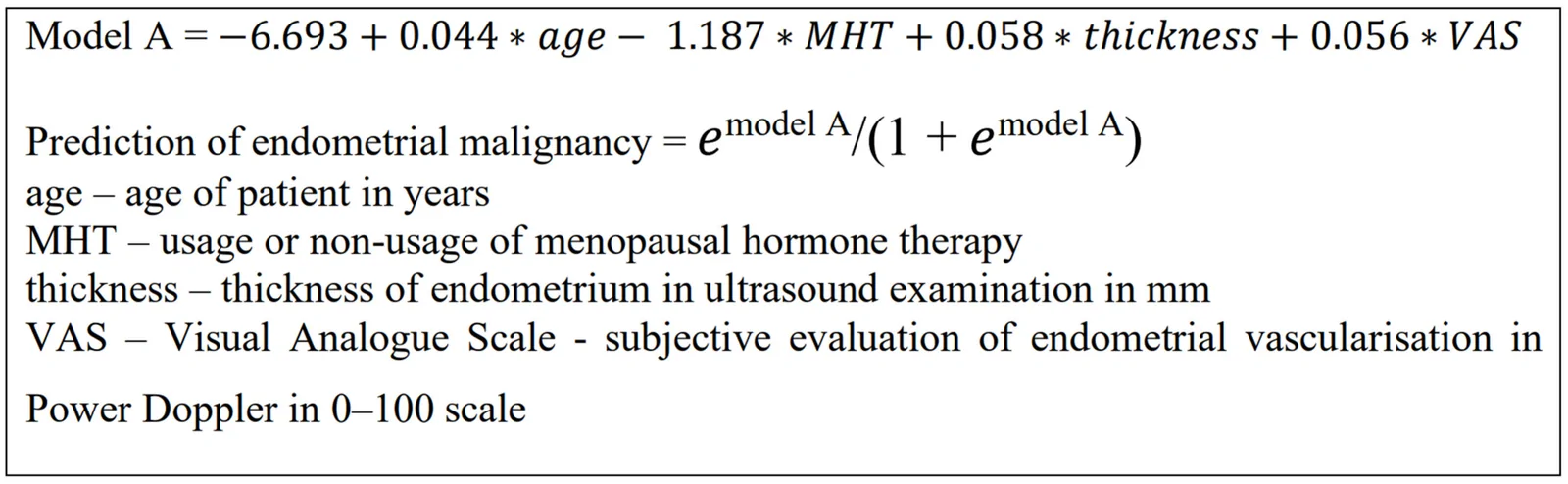
Model A.

Sladkevicius and Valentin subsequently evaluated mathematical models for predicting endometrial malignancy among women with postmenopausal bleeding and endometrial thickness ≥ 4.5 mm [22]. Model A combines age, menopausal hormone therapy use, endometrial thickness, and a subjective assessment of endometrial vascularization (Figure 2). Its performance in a broader referral population, including premenopausal women, remains uncertain. We therefore evaluated HE4, REM and Model A in women undergoing histological assessment for suspected endometrial pathology.

## 2. Materials and Methods

A total of 110 patients treated in the Department of Operative Gynaecology and Gynaecological Oncology of Adults and Adolescents of Pomeranian Medical University between year 2017 and 2020 because of abnormal uterine bleeding or ultrasound endometrial abnormalities were enrolled in this prospective study. Inclusion criteria were age ≥ 18, ultrasound endometrial abnormalities (endometrial thickness ≥ 4 mm, suspicion of endometrial polyp or presence of fluid in the uterine cavity) or presence of abnormal uterine bleeding. Exclusion criteria were intake of tamoxifen, severe comorbidities including heart, renal, or hepatic failure and non-epithelial malignancy of uterus in the final histopathological examination.

Each patient admitted to the clinic had a detailed anamnesis concerning age, parity, usage of hormonal menopausal therapy, BMI, comorbidities, medicaments, absence or presence of abnormal uterine bleeding according to the ACOG criteria [23]. Every patient had then undergone physical examination comprising transvaginal ultrasound with evaluation of the endometrial thickness performed on a GE Voluson E8 ultrasound system (GE Healthcare Austria GmbH & Co OG, Zipf, Austria) using an endovaginal transducer in the Routine HI setting. On the day of admission, the patients had their blood sample taken in order to determine HE4 and CA125 marker levels. HE4 serum concentration was measured using Elecsys ECLIA test by Roche Diagnostics GmbH, Mannheim, Germany. Hysteroscopy with histopathological material sampling was performed the next day after admission. 

All examinations were performed by the same operator and were carried out in a predetermined manner.

Statistical analyses for the original study were performed in R (version 4.2.2). The cohort comprised 18 women with histologically confirmed endometrial cancer and 92 without malignancy. Assessed variables included age, abnormal uterine bleeding, menopausal hormone therapy, BMI, hypertension, diabetes, risk-factor burden, parity, serum HE4, ultrasound endometrial thickness, and vascularization. The histopathological examination served as the diagnostic reference standard. Post hoc analyses were additionally performed on the available individual-level study dataset using Python 3.13.5 (SciPy, scikit-learn and statsmodels).

The original analyses used univariable logistic regression and the Wilcoxon–Mann–Whitney test, and presented empirical ROC curves for HE4, REM and Model A. Scores for REM and Model A were taken from the original study dataset and were not refitted or recalibrated during this revision; the published equations are shown in Figure 1 and Figure 2. In the additional post hoc analysis, AUCs were estimated from individual observations, with stratified percentile bootstrap 95% confidence intervals (5000 resamples). Cut-offs were selected by the maximum Youden index within this same cohort. Sensitivity, specificity, positive predictive value (PPV) and negative predictive value (NPV) were calculated from the corresponding 2 × 2 tables. Paired AUCs were compared using the DeLong method. An exploratory logistic regression included age (years), endometrial thickness (mm) and log2-transformed HE4, so that the HE4 odds ratio denotes a doubling of concentration. Analyses were two-sided, with *p* < 0.05 considered statistically significant; all additional cut-off and multivariable findings are exploratory and internally unvalidated. IQR denotes interquartile range. As a supplementary threshold-transportability analysis, the original published REM (0.3185) and Model A (0.51) cut-offs were applied without recalibration or threshold optimization, and their classification performance was compared descriptively with the cohort-derived Youden cut-offs. The published model equations are reproduced in editable form in Figure 1 and Figure 2.

## 3. Results

Clinical characteristics of the study group are presented in Table 1. The median age was 61 years. Abnormal uterine bleeding was reported in 29% of patients; 43% had normal BMI and 30% were obese. More than half had hypertension and 17% had diabetes. Endometrial cancer was identified in 18 of 110 women (16.4%), whereas 92 (83.6%) had non-malignant findings, including 30 with histologically normal endometrium.

**Figure 3:**
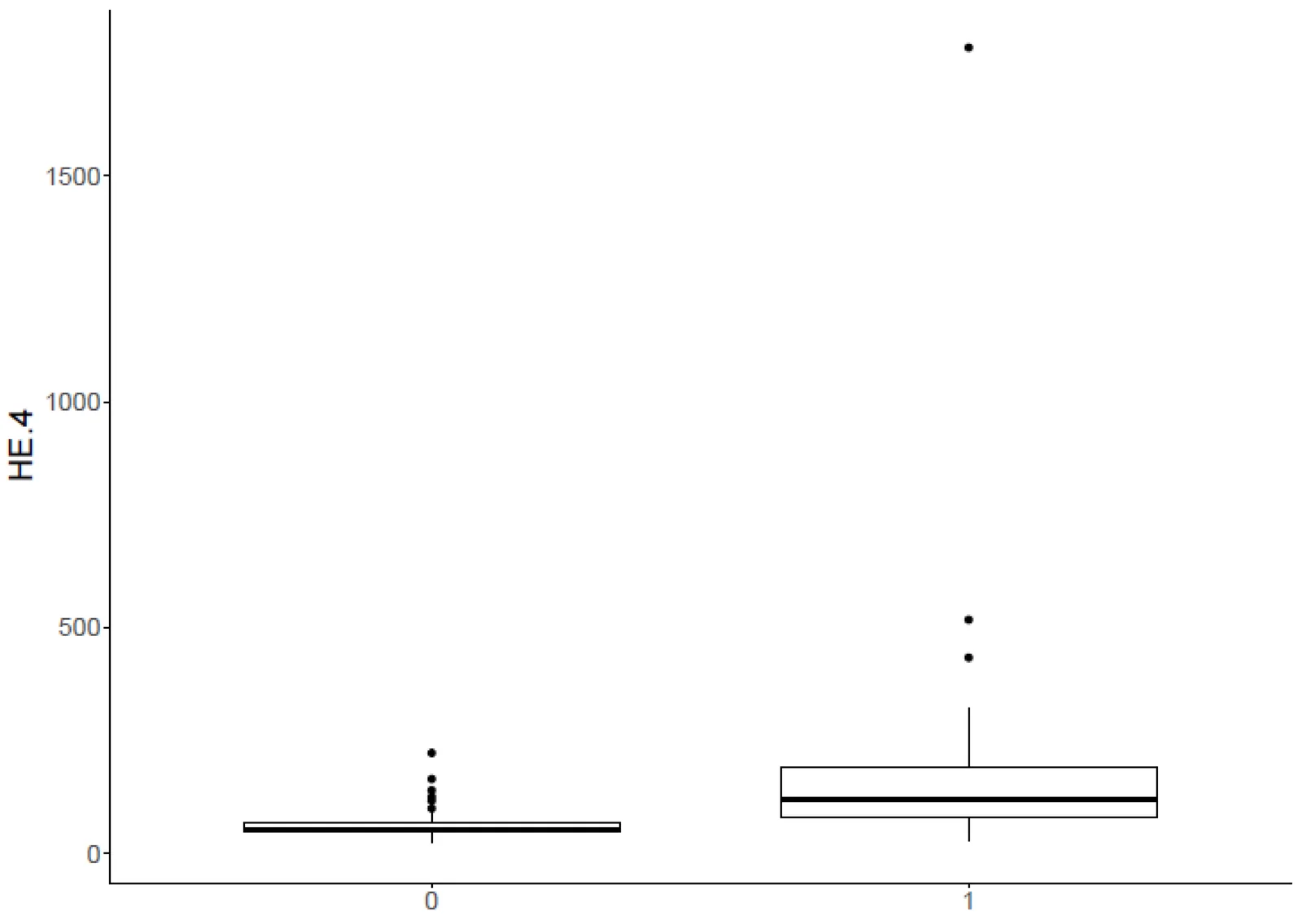
Serum HE4 concentration in patients with non-malignant findings (0) and endometrial cancer (1).

Patients with endometrial cancer had significantly higher HE4 serum marker levels as compared to ones with benign lesions (*p* < 0.001). Median of serum HE4 concentration in the malignant lesion group was 118.35 pmol/L (IQR 110.72 pmol/L), whereas in benign lesion group 52.3 pmol/L (IQR 20.35 pmol/L) (Figure 3).

In an additional post hoc three-group analysis, median serum HE4 was 55.25 pmol/L in women with histologically normal endometrium (n = 30), 51.60 pmol/L in women with non-malignant endometrial lesions (n = 62), and 118.35 pmol/L in women with cancer (n = 18). The overall difference was significant (Kruskal–Wallis *p* < 0.001). Pairwise comparisons showed higher HE4 in cancer than in either non-malignant subgroup (Mann–Whitney *p* < 0.001 for each unadjusted comparison), but not between the two non-malignant subgroups (*p* = 0.168). These groups were formed for descriptive analysis and do not include healthy volunteers.

**Figure 4:**
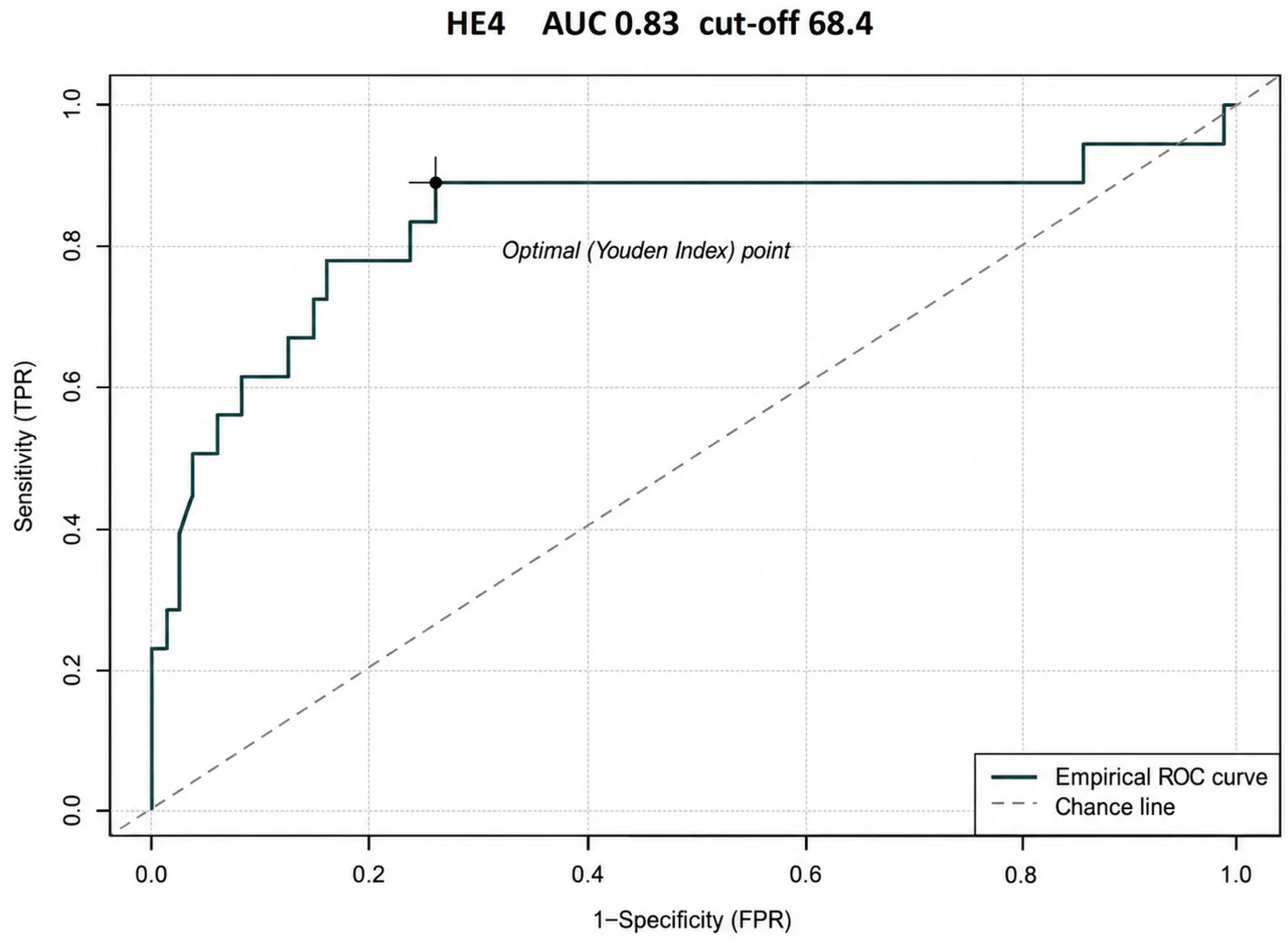
Empirical receiver operating characteristic (ROC) curve for serum HE4 in the differentiation of endometrial cancer (n = 18) from non-malignant findings (n = 92). The original curve displays the sample-derived Youden threshold; confidence intervals and classification metrics from the reanalysis are given in Table 2.

Empirical ROC analysis was performed for HE4 (Figure 4); full post hoc diagnostic accuracy estimates are presented with those of REM and Model A in Table 2.

Univariable logistic regression revealed that patients with hypertension, type 2 diabetes or 3 risk factors had significantly elevated risk of endometrial cancer occurrence. Patients who gave birth at least once had a lower risk of endometrial cancer (Table 3).

**Figure 5:**
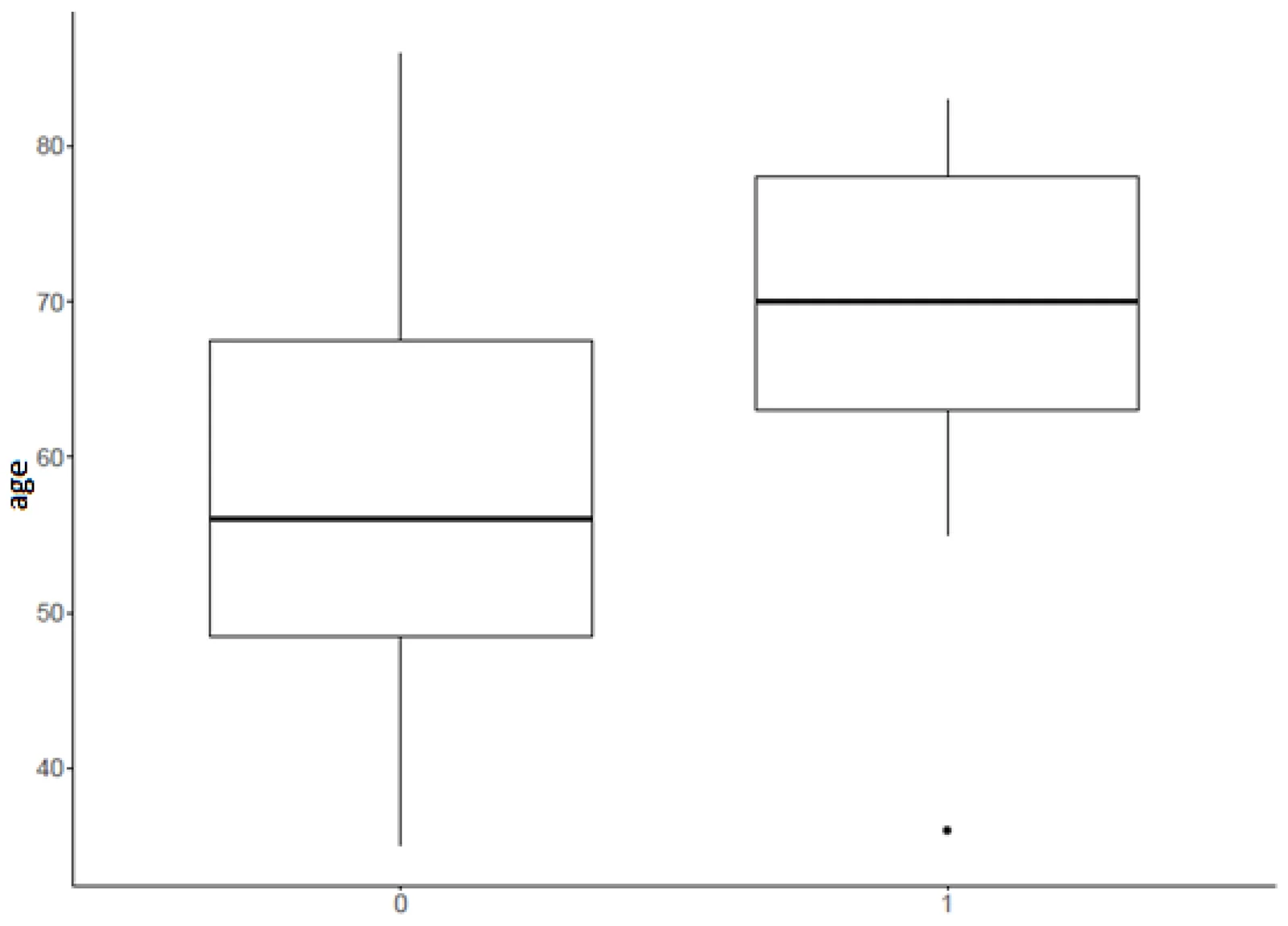
Age of patients with non-malignant findings (0) and endometrial cancer (1).

Endometrial cancer patients differed from the benign lesions patients by age (*p* < 0.001). Median age in endometrial cancer group was 70 (IQR 15) and in benign lesion group 56 (IQR 19) (Figure 5).

**Figure 6:**
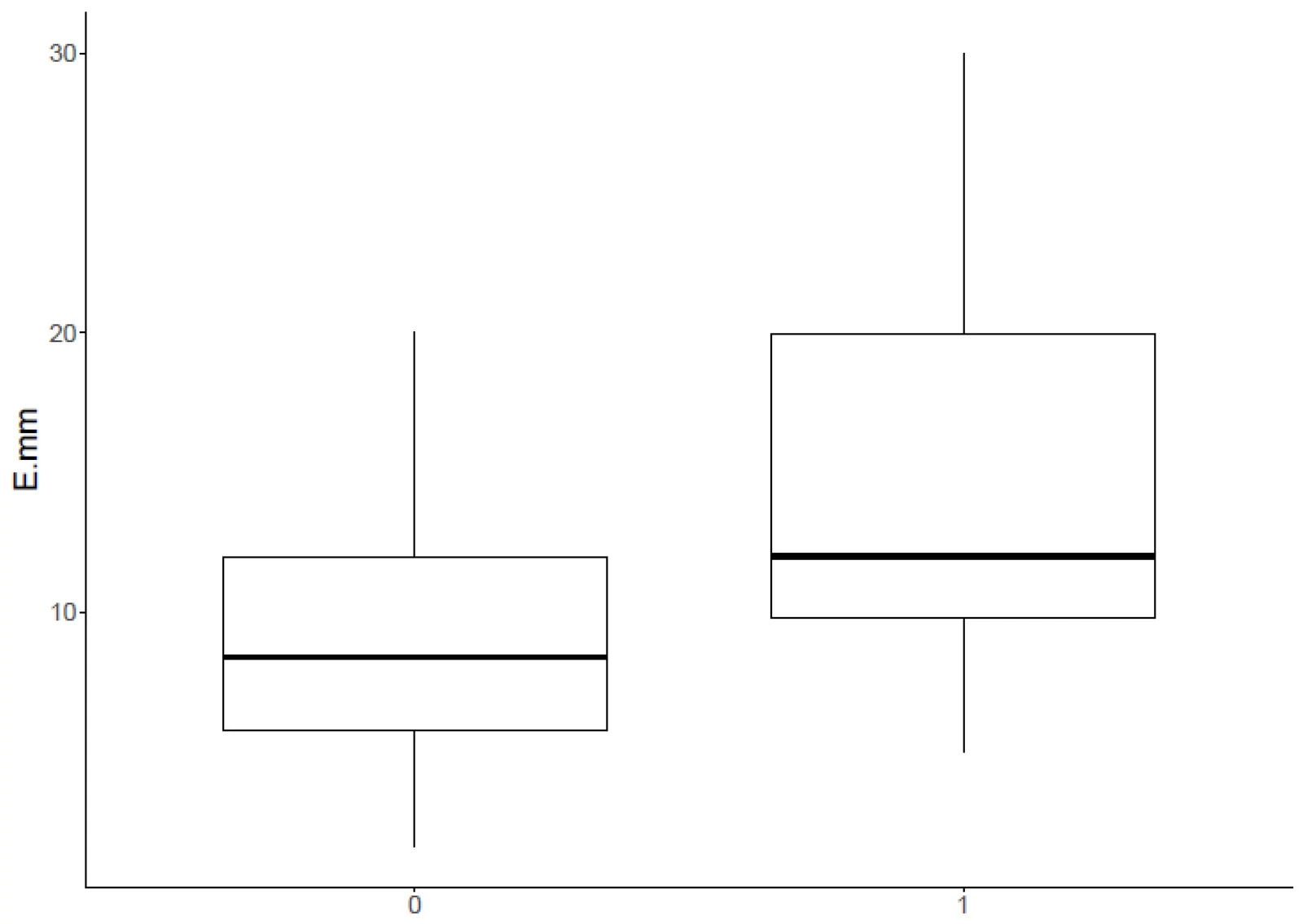
Endometrial thickness in patients with non-malignant findings (0) and endometrial cancer (1).

Patients with endometrial cancer had a significantly thicker endometrium compared to patients with benign lesions (*p* < 0.001). The median in the malignant lesion group was 12 mm (IQR 10.8), whereas in the benign lesion group it was 8.4 mm (IQR 6.2) (Figure 6).

**Figure 7:**
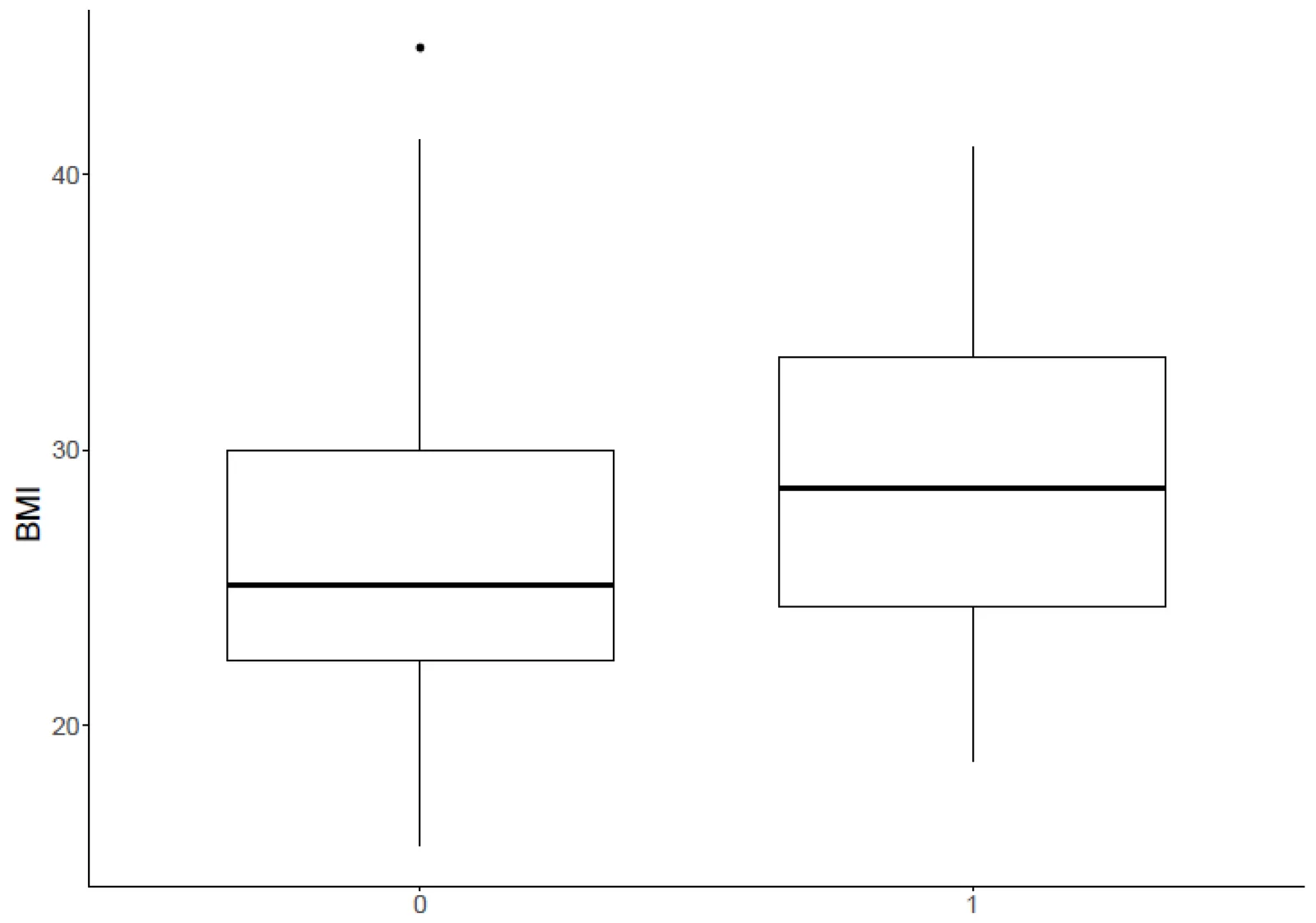
BMI in patients with non-malignant findings (0) and endometrial cancer (1).

Patients diagnosed with endometrial cancer had a higher BMI (28.6 [IQR 9.09]), compared with women without diagnosed cancer (25.1 [IQR 7.76]). This observation was at the verge of statistical significance (*p* = 0.086) (Figure 7).

Risk of Endometrial Malignancy (REM) scoring was calculated using the published formula (Figure 1). In the original univariable analysis, a REM score between 0.35 and 1.00 was associated with higher odds of cancer (OR 23.7; 95% CI 4.57–436; *p* = 0.003) than a score between 0.002 and 0.33. Model A was calculated using its published formula (Figure 2). Model A scores from 0.03 to 0.62 were associated with higher odds of cancer than scores from 0.00 to 0.03 (OR 6.37; 95% CI 1.94–28.9; *p* = 0.005) (Table 4). These historical, cohort-median comparisons differ from the Youden-derived ROC thresholds reported in Table 4.

**Figure 8:**
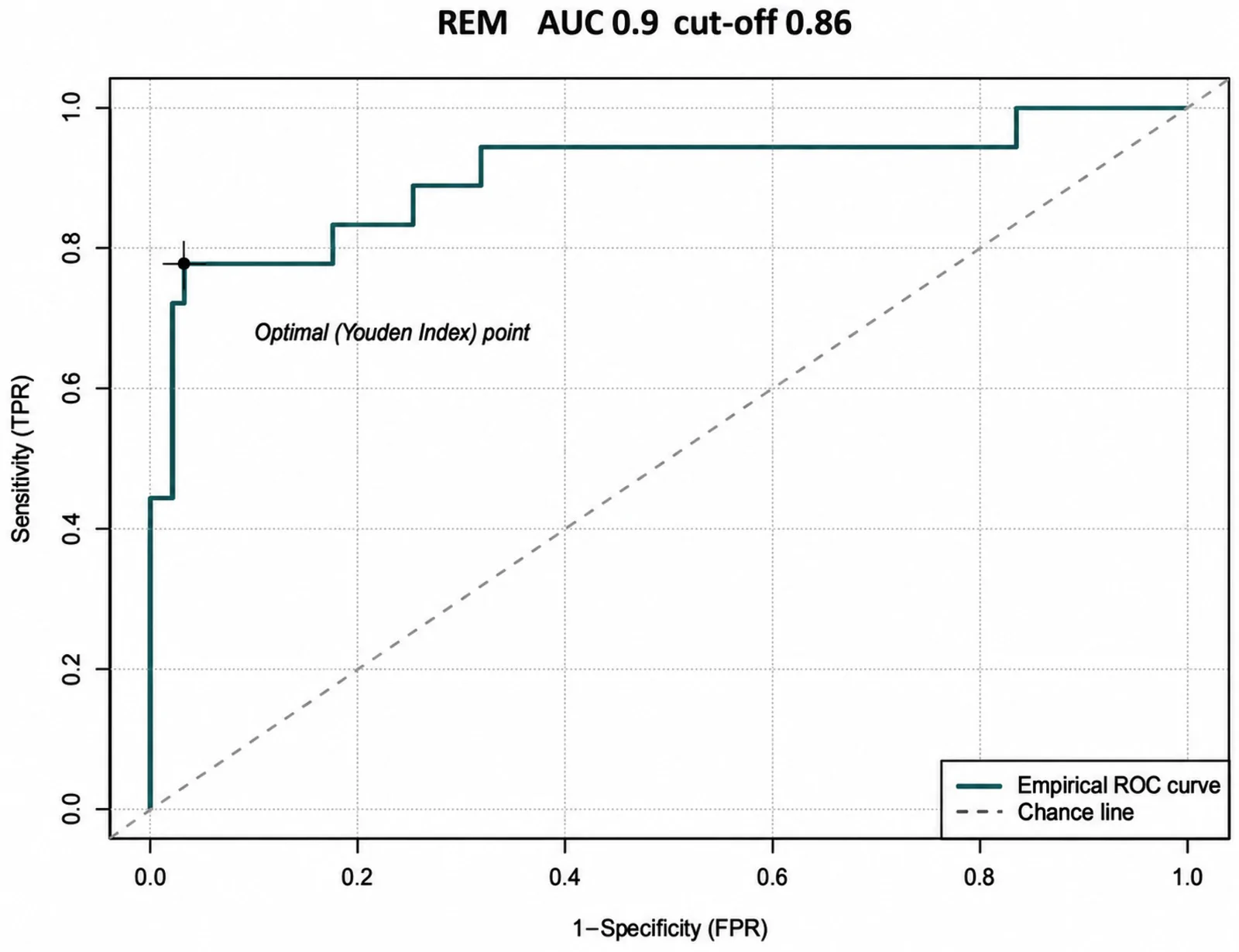
Empirical receiver operating characteristic (ROC) curve for the original REM score in distinguishing endometrial cancer from non-malignant findings (18 versus 92 women). The indicated threshold was optimized in this cohort; detailed diagnostic metrics are shown in Table 2.

**Figure 9:**
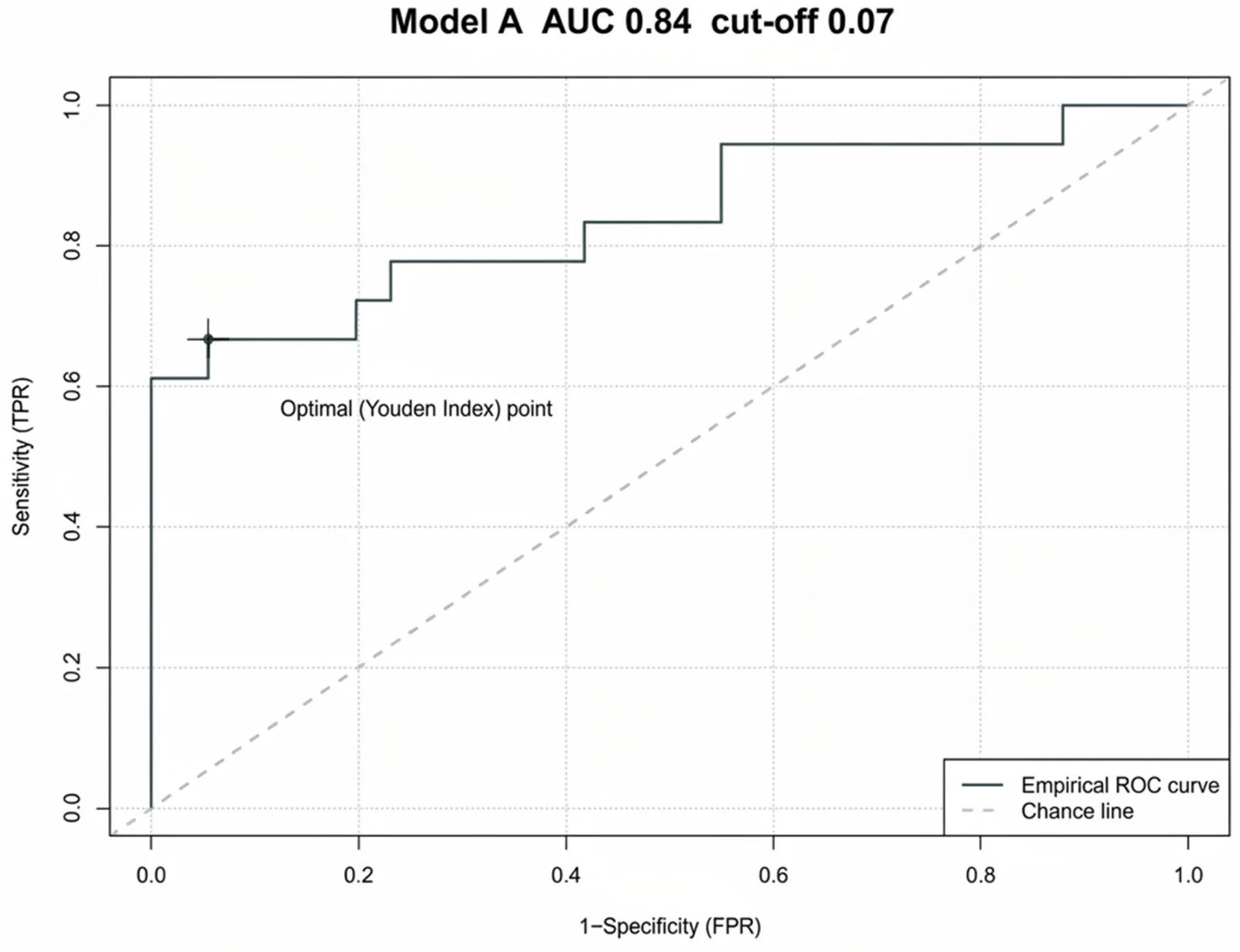
Empirical receiver operating characteristic (ROC) curve for the original Model A score in distinguishing endometrial cancer from non-malignant findings (18 versus 92 women). The indicated threshold was optimized in this cohort; detailed diagnostic metrics are shown in Table 2.

Empirical ROC curves were also drawn for REM and Model A (Figure 8 and Figure 9). The three methods were compared using the paired DeLong test as described below.

On reanalysis of all 110 individual records, the AUCs were 0.834 for HE4, 0.905 for REM and 0.840 for Model A (Table 2). The corresponding sample-selected thresholds were 68.4 pmol/L, 0.86307206 and 0.07386795. The latter values are displayed with sufficient precision to retain the exact observed classifications at the thresholds. Table 2 provides the associated classification counts and performance measures. Because the same sample was used to select and evaluate these thresholds, the estimates are apparent (non-validated) diagnostic performance.

AUC confidence intervals were obtained with stratified percentile bootstrap resampling (5000 replicates). Se, sensitivity; Sp, specificity; PPV, positive predictive value; NPV, negative predictive value; TP, true positive; FN, false negative; TN, true negative; FP, false positive. PPV and NPV pertain to the 16.4% malignancy prevalence in this cohort.

In paired DeLong tests, the differences in AUC were not statistically significant: REM versus HE4, *p* = 0.106; REM versus Model A, *p* = 0.108; and HE4 versus Model A, *p* = 0.933. The numerically greater REM AUC therefore cannot be interpreted as evidence of statistically superior accuracy.

In an exploratory post hoc multivariable logistic regression model adjusting for age and endometrial thickness, log2-transformed HE4 remained associated with endometrial cancer (adjusted OR per doubling, 5.41; 95% CI 1.81–16.21; *p* = 0.0025; Table 5). This result does not establish a validated independent diagnostic test.

The original REM threshold identified 17/18 cancers but generated 42 false-positive classifications among 92 women without cancer. The original Model A threshold identified only 2/18 cancers (16 false negatives), although no false positives occurred in this small sample. Cohort-optimized cut-offs and their performance are exploratory. TP, true positive; FN, false negative; TN, true negative; FP, false positive; PPV, positive predictive value; NPV, negative predictive value.

**Figure 10:**
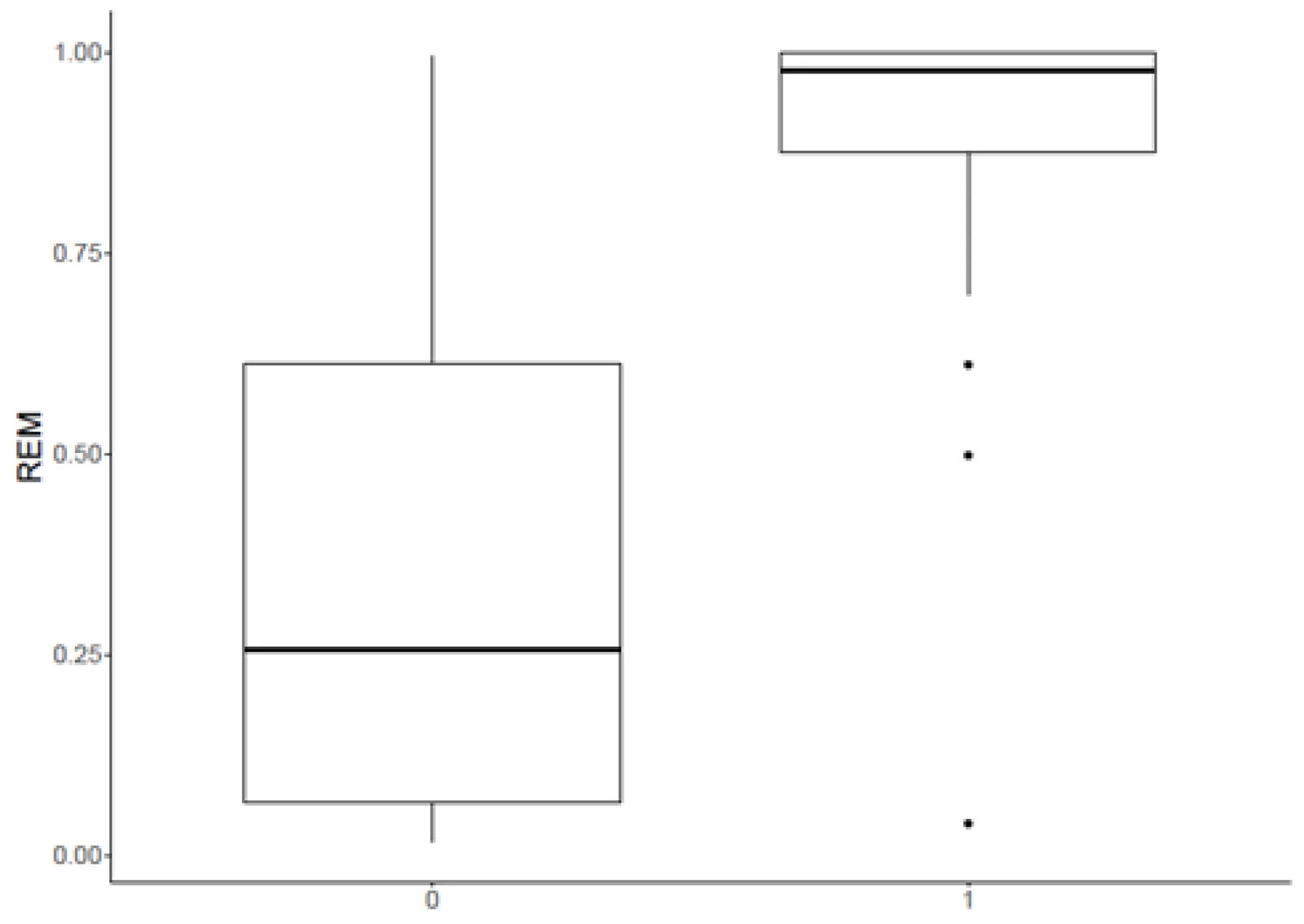
REM in patients with non-malignant findings (0) and endometrial cancer (1).

Statistical analysis using Wilcoxon test showed a significant difference in REM score between patients with benign and malignant lesions (*p* < 0.001). Median for cancer diagnosed patients was 0.98 (IQR 0.13) and for patients without endometrial cancer 0.26 (IQR 0.54) (Figure 10).

**Figure 11:**
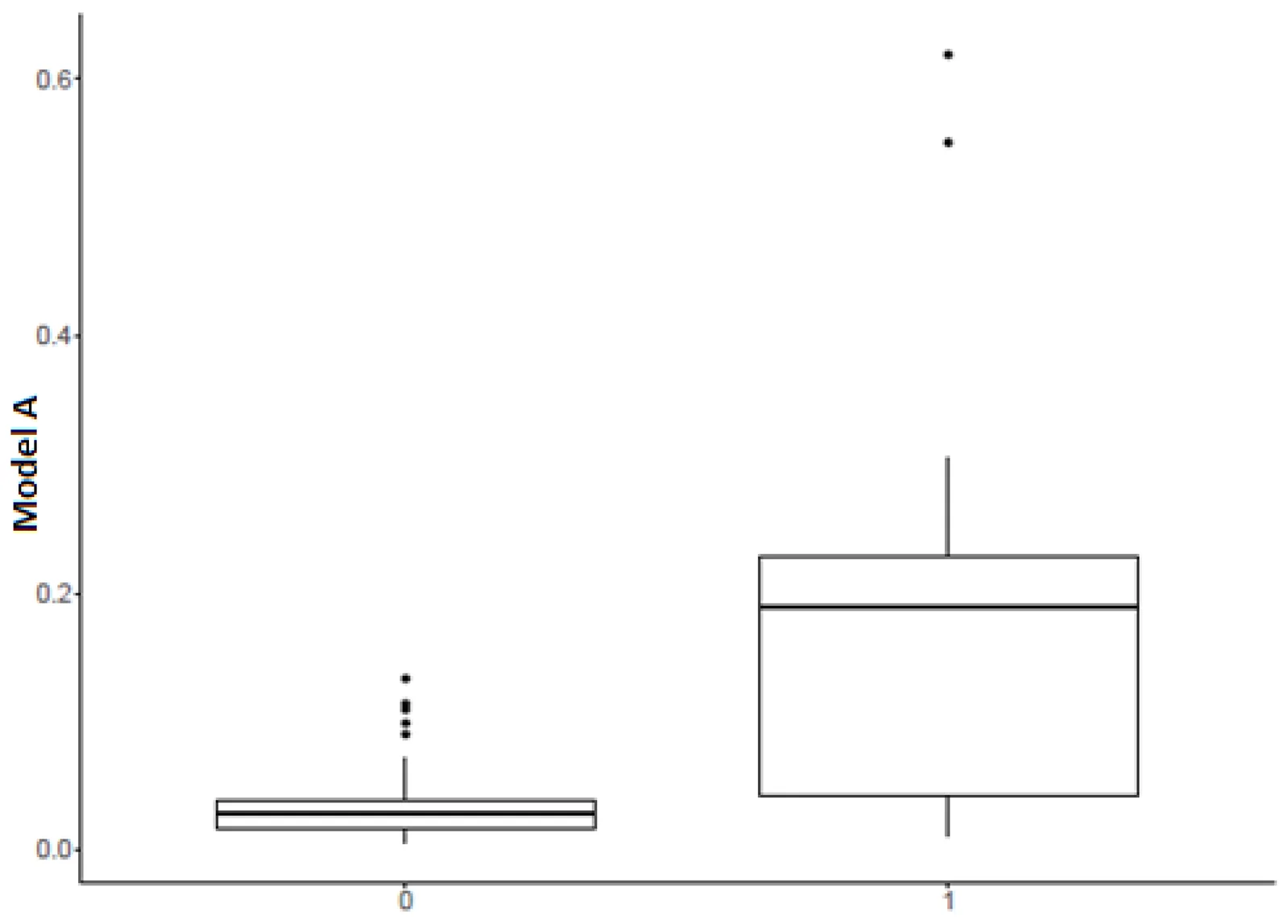
Model A in patients with non-malignant findings (0) and endometrial cancer (1).

Model A was also analysed with Wilcoxon test, which also revealed a statistically significant difference between patients with malignant and benign lesions (*p* < 0.001). Median result in endometrial cancer group was 0.19 (IQR 0.19) and in group without endometrial cancer 0.003 (IQR 0.02) (Figure 11).

## 4. Discussion

In this prospective cohort of 110 women referred for endometrial assessment, the central findings were that serum HE4 was substantially higher in patients with endometrial cancer than in those without malignancy, that HE4 remained associated with cancer in an exploratory model accounting for age and endometrial thickness, and that the published REM and Model A scores discriminated malignant from non-malignant findings. REM had the highest numerical AUC, but its advantage over HE4 alone or Model A was not statistically significant. Applying the original published thresholds also revealed important differences between model discrimination and actual classification performance in this cohort.

The median serum HE4 concentration was 118.35 pmol/L in the cancer group and 52.3 pmol/L in women without malignancy (*p* < 0.001). This accords with earlier reports of increased HE4 in endometrial cancer, including pooled analyses showing moderate sensitivity and comparatively high specificity [12,15,16,23]. In our supplementary three-group analysis, HE4 was higher in the cancer group than both the histologically normal and non-malignant lesion groups, whereas the two groups without cancer did not differ significantly. These comparisons reflect symptomatic or sonographically selected women, not a healthy population-screening sample.

In an exploratory post hoc multivariable model, a doubling of HE4 concentration was associated with higher odds of cancer after adjustment for age and sonographic endometrial thickness (adjusted OR 5.41; 95% CI 1.81–16.21; *p* = 0.0025). This may indicate that HE4 provides diagnostic information beyond these two readily available parameters. However, only 18 malignant cases were included, the confidence interval was wide, and the model was neither internally nor externally validated. The result should therefore be regarded as hypothesis-generating rather than evidence of a clinically validated independent predictor.

For the three evaluated approaches, the AUCs were 0.834 for HE4, 0.905 for REM, and 0.840 for Model A. All three methods showed discriminatory ability within the enrolled population. Nevertheless, paired DeLong comparisons did not demonstrate significant differences (REM versus HE4, *p* = 0.106; REM versus Model A, *p* = 0.108; HE4 versus Model A, *p* = 0.933). An apparently higher AUC cannot on its own establish the superiority of one model. Similarly, the sensitivity and specificity achieved at cut-offs selected with the Youden index in the same dataset represent apparent, rather than validated, diagnostic accuracy.

The behavior of the originally published cut-offs is particularly informative (Table 6). At the original REM threshold of 0.3185 [18], sensitivity was 94.4% (17/18) but specificity was only 54.3% (50/92), compared with 77.8% and 96.7%, respectively, at the cohort-derived cut-off of 0.86307206. In contrast, at the original Model A threshold of 0.51 [21], sensitivity was only 11.1% (2/18), despite an observed specificity of 100% (92/92). At the cohort-derived Model A threshold of 0.07386795, sensitivity and specificity were 66.7% and 94.6%. These sample-based estimates indicate marked threshold transportability problems, especially for Model A, but they are imprecise because the cancer subgroup was small. The 100% specificity at the original Model A threshold should not be interpreted as proof of perfect specificity in other cohorts.

Differences between the original development populations and our cohort may partly explain these findings. In particular, Model A was developed and subsequently evaluated in women with postmenopausal bleeding and sonographic endometrial thickness ≥ 4.5 mm [21,24]; our cohort also included premenopausal women and patients with other referral indications. Unlike the REM score, Model A additionally relies on a subjective vascularization assessment, for which interobserver agreement is a potential concern [25]. Published evaluations of REM and its obesity-inclusive extension REM-B have reported encouraging performance [18,26,27,28], but those estimates should not be transferred automatically to a different patient spectrum or to newly optimized decision thresholds.

Clinically, a marker-based model could potentially assist risk stratification and prioritization of diagnostic evaluation, but the present results do not establish a safe threshold for withholding tissue sampling. At the sample-derived cut-offs, HE4, REM, and Model A missed two, four, and six cancer cases, respectively. Neither HE4 nor the algorithms are specific enough or sufficiently validated to replace histopathological assessment. Future work should prospectively evaluate the original coefficients and thresholds in adequately powered cohorts and examine calibration, discrimination, and clinical utility before recommending risk-adapted pathways.

Several associations involving conventional clinical factors were consistent with prior epidemiological reports. Hypertension (unadjusted OR 3.79; 95% CI 1.31–12.7; *p* = 0.019), type 2 diabetes (OR 5.82; 95% CI 1.88–18.2; *p* = 0.002), and the concurrence of three metabolic risk factors (OR 6.00; 95% CI 1.69–21.3; *p* = 0.005) were associated with greater odds of finding cancer in our referral population. The relationships of metabolic syndrome, diabetes, and hypertension with incident endometrial cancer have been described in larger observational syntheses [9,10,29,30,31,32,33]. Our associations are unadjusted diagnostic comparisons and should not be interpreted as estimates of population incidence risk. BMI was numerically greater in the cancer group (28.6 versus 25.1 kg/m^2^), but the difference did not reach statistical significance (*p* = 0.086).

Previous childbirth was associated with lower odds of endometrial cancer in our cohort (OR 0.22; 95% CI 0.06–0.82; *p* = 0.020), consistent with pooled evidence for an inverse relationship between parity and endometrial cancer risk [34,35,36]. Endometrial thickness ≥ 9 mm was associated with greater odds of malignancy (OR 4.27; 95% CI 1.42–16.0; *p* = 0.016). Interpretation of any thickness threshold requires the clinical context, especially menopausal status and bleeding, as reflected in the endometrial ultrasound literature [24,37,38,39]. Because our cohort included premenopausal women, this threshold should not be generalized as a universal diagnostic rule.

HE4 is not an endometrium-specific marker: it can be elevated in other malignancies and may be influenced by age or renal function. Experimental studies suggest associations of HE4 with proliferation and invasion in endometrial cancer models, but these mechanistic data do not establish causation for the serum findings in our study [40,41]. Endometrial cancer is biologically heterogeneous, including molecularly defined groups [42], yet the present analysis addressed diagnosis rather than tumor subtype, prognosis, or treatment response. The methodological limitations of this interpretation are detailed below.

### Study Limitations

This study has several limitations. It was conducted at a single center and included only 18 women with endometrial cancer, resulting in wide confidence intervals and limiting the reliability of multivariable models. The added regression analysis was exploratory, included three clinically selected covariates, and was not internally or externally validated; residual confounding, model instability, and optimism in apparent performance remain possible. The Youden thresholds were selected and assessed in the same dataset and should not be interpreted as clinically established decision limits. The observed PPV and NPV also reflect the relatively high cancer prevalence (16.4%) of this selected referral cohort.

The study did not include a separate group of asymptomatic healthy volunteers, although 30 patients had histologically normal endometrium. The source dataset did not support a reliable comparison of CA125 or additional molecular markers across all 110 women, and complete information on previous oncological treatment, tumor stage distribution, and renal function was not available for validated adjustment. The cohort also included women outside the original eligibility setting of Model A (postmenopausal bleeding and endometrial thickness ≥ 4.5 mm). Consequently, these results cannot be generalized to population screening or used to change diagnostic pathways without prospective multicenter validation.

## 5. Conclusions

In this prospective cohort of 110 women undergoing endometrial investigation, serum HE4, endometrial thickness and the published REM and Model A scores showed associations with histologically diagnosed endometrial cancer. The AUCs were 0.834 for HE4, 0.905 for REM and 0.840 for Model A; although REM ranked highest numerically, its AUC did not differ significantly from those of the other approaches. In an exploratory adjusted analysis, HE4 remained associated with malignancy after accounting for age and endometrial thickness.

These findings support further evaluation of biochemical, clinical and ultrasound variables as complementary tools for diagnostic risk stratification, not as substitutes for histopathological assessment. Given the small cancer group, post hoc selection of thresholds and absence of independent validation, larger prospective studies are required before routine clinical use.

## Figures and Tables

**Table 1:** The clinical characteristics of the patients enrolled.

PARAMETER	MEDIAN	IQR
Age [years]	61	18.75
BMI [kg/m^2^] (*n*)		
18.5–24.99	48	
25–29.99	29	
≥30	33	
PARAMETER	YES	NO
Abnormal uterine bleeding	32	78
Hypertension	59	51
Diabetes	19	91
≥3 risk factors	12	98
Previous cancer (non-endometrial) in anamnesis	9	101
Type of endometrial lesion (n)		
Normal endometrium	30	
Polyp	58	
Hyperplasia	4	
Cancer	18	

**Table 2:** Post hoc diagnostic accuracy of serum HE4, REM and Model A for endometrial cancer versus non-malignant findings (n = 18 vs. n = 92). Cut-offs maximize the Youden index in this dataset.

Method	Cut-Off	AUC (95% CI)	TP/FN/TN/FP	Se (%)	Sp (%)	PPV (%)	NPV (%)
HE4	68.4 pmol/L	0.834 (0.686–0.951)	16/2/69/23	88.9	75.0	41.0	97.2
REM	0.86307206	0.905 (0.794–0.981)	14/4/89/3	77.8	96.7	82.4	95.7
Model A	0.07386795	0.840 (0.702–0.948)	12/6/87/5	66.7	94.6	70.6	93.5

**Table 3:** Univariable logistic regression for selected clinical features in the whole cohort (18 cancer cases and 92 non-malignant findings). Odds ratios compare the category of interest with the stated reference category and are unadjusted.

PARAMETER	Compared Category	Reference Category	OR	95% CI	*p*
AUB	YES	NO	2.23	0.77–6.34	0.13
BMI [kg/m^2^]	Obesity	overweight/normal	1.88	0.63–5.37	0.2
PARITY	YES	NO	0.22	0.06–0.82	0.02
HYPERTENSION	YES	NO	3.79	1.31–12.7	0.019
TYPE 2 DIABETES	YES	NO	5.82	1.88–18.2	0.002
3 RISK FACTORS	YES	NO	6.0	1.69–21.3	0.005

**Table 4:** Univariable logistic regression results for REM and Model A, comparing higher versus lower score subgroups defined by the sample median. The medians and ranges describe the score distributions in the two subgroups.

PARAMETER	Median of 1. Subgroup (Range)	Median of 2. Subgroup (Range)	OR	95% CI	*p*
**REM**	0.11 (0.002–0.33)	0.72 (0.35–1.00)	23.7	4.57–436	0.003
**Model A**	0.02 (0.00–0.03)	0.10 (0.03–0.62)	6.37	1.94–28.9	0.005

**Table 5:** Exploratory multivariable logistic regression for histologically confirmed endometrial cancer (18 cases, 92 non-malignant findings).

Variable	Adjusted OR	95% CI	*p*
Age, per 1 year	1.05	0.97–1.13	0.203
Endometrial thickness, per 1 mm	1.23	1.08–1.41	0.0016
HE4, per doubling of concentration	5.41	1.81–16.21	0.0025

OR, odds ratio; CI, confidence interval. Coefficients were estimated in the same cohort used for descriptive analyses; no external or internal validation was performed.

**Table 6:** Classification performance at the published REM and Model A cut-offs compared with the cut-offs selected by the Youden index in the current cohort (18 cancer cases, 92 non-malignant findings).

Score/Threshold	Type of Cut-Off	Cut-Off	TP/FN/TN/FP	Sensitivity (%)	Specificity (%)	PPV (%)	NPV (%)
REM	Published	0.3185	17/1/50/42	94.4	54.3	28.8	98.0
REM	Cohort Youden	0.86307206	14/4/89/3	77.8	96.7	82.4	95.7
Model A	Published	0.51	2/16/92/0	11.1	100.0	100.0	85.2
Model A	Cohort Youden	0.07386795	12/6/87/5	66.7	94.6	70.6	93.5

## Data Availability

The data presented in this study are not publicly available due to privacy and ethical restrictions concerning the protection of patient data. The data may be available from the corresponding author upon reasonable request, subject to appropriate ethical and data protection requirements.
